# Role of Epidermal Growth Factor Receptor (EGFR) and Its Ligands in Kidney Inflammation and Damage

**DOI:** 10.1155/2018/8739473

**Published:** 2018-12-23

**Authors:** Sandra Rayego-Mateos, Raul Rodrigues-Diez, Jose Luis Morgado-Pascual, Floris Valentijn, Jose M. Valdivielso, Roel Goldschmeding, Marta Ruiz-Ortega

**Affiliations:** ^1^Vascular and Renal Translational Research Group, Institut de Recerca Biomèdica de Lleida (IRBLleida), Lleida 25198, Spain; ^2^IdiPAZ, Madrid, Spain; ^3^Cellular Biology in Renal Diseases Laboratory, Universidad Autónoma Madrid, IIS-Fundación Jiménez Díaz, Madrid, Spain; ^4^University Medical Center Utrecht, Utrecht, Netherlands

## Abstract

Chronic kidney disease (CKD) is characterized by persistent inflammation and progressive fibrosis, ultimately leading to end-stage renal disease. Although many studies have investigated the factors involved in the progressive deterioration of renal function, current therapeutic strategies only delay disease progression, leaving an unmet need for effective therapeutic interventions that target the cause behind the inflammatory process and could slow down or reverse the development and progression of CKD. Epidermal growth factor receptor (EGFR) (ERBB1), a membrane tyrosine kinase receptor expressed in the kidney, is activated after renal damage, and preclinical studies have evidenced its potential as a therapeutic target in CKD therapy. To date, seven official EGFR ligands have been described, including epidermal growth factor (EGF) (canonical ligand), transforming growth factor-*α*, heparin-binding epidermal growth factor, amphiregulin, betacellulin, epiregulin, and epigen. Recently, the connective tissue growth factor (CTGF/CCN2) has been described as a novel EGFR ligand. The direct activation of EGFR by its ligands can exert different cellular responses, depending on the specific ligand, tissue, and pathological condition. Among all EGFR ligands, CTGF/CCN2 is of special relevance in CKD. This growth factor, by binding to EGFR and downstream signaling pathway activation, regulates renal inflammation, cell growth, and fibrosis. EGFR can also be “transactivated” by extracellular stimuli, including several key factors involved in renal disease, such as angiotensin II, transforming growth factor beta (TGFB), and other cytokines, including members of the tumor necrosis factor superfamily, showing another important mechanism involved in renal pathology. The aim of this review is to summarize the contribution of EGFR pathway activation in experimental kidney damage, with special attention to the regulation of the inflammatory response and the role of some EGFR ligands in this process. Better insights in EGFR signaling in renal disease could improve our current knowledge of renal pathology contributing to therapeutic strategies for CKD development and progression.

## 1. Introduction

Chronic kidney disease (CKD) is a devastating progressive disease that affects 5–7% of the world's population. CKD has become a public health priority due to the increasing incidence of type 2 diabetes mellitus, hypertension, obesity, and aging [1]. CKD is characterized by diverse insults that trigger persistent inflammation, development of fibrosis, and loss of renal function ultimately leading to end-stage renal disease. Nowadays, the therapeutic protocols applied for the treatment against CKD have limited effectiveness underscoring the importance of the development of new molecular diagnostic approaches and therapeutic targets to either prevent or delay the progression of renal diseases.

Many preclinical studies have shown that the epidermal growth factor receptor (EGFR) can be a potential therapeutic target for renal diseases, as we will review here. Activation of the EGFR signaling pathway is linked to the regulation of several cellular responses, including cell proliferation, inflammatory processes, and extracellular matrix regulation—all of them being involved in the onset and progression of renal damage. Nowadays, there are seven official EGFR ligands, including the following: EGF (canonical ligand), transforming growth factor-*α* (TGFA), heparin-binding EGF-like growth factor (HBEGF), amphiregulin (AREG), betacellulin (BTC), epiregulin (EPR), and epigen (EPGN) [2–7]. Recently, the connective tissue growth factor (CTGF/CCN2) has been described as a novel EGFR ligand [7]. Among all EGFR ligands, CTGF has been considered as a therapeutic target and a potential biomarker of human renal diseases [8–15]. The aim of this review is to summarize the contribution of EGFR pathway activation in experimental kidney damage, with special attention to the regulation of the inflammatory response and the role of some EGFR ligands in this process.

### 1.1. The EGFR Activation Pathways

The binding of neurotransmitters, hormones, or growth factors (ligands) to their membrane receptors produces biochemical changes inside the cell, which lead to a specific response to the initial stimulus. There are different groups of membrane receptors, all defined by their signal transduction mechanisms; these include ionotropic receptors, G protein-coupled receptors (GPCRs), and receptors with tyrosine kinase (RTK) activity. The EGFR (also known as HER1; ERBB1) is a transmembrane glycoprotein of 1186 aa (180 KDa) that belongs to the ERBB family of tyrosine kinase receptors, which is composed of members such as HER2/neu (ERBB2), HER3 (ERBB3), and HER4 (ERBB4). EGFR comprises a cysteine-rich extracellular domain (responsible for ligand binding), a transmembrane domain, and an intracellular domain with tyrosine kinase regions (activation domain) [16]. In most cases, EGFR is activated either directly or indirectly, by EGFR transactivation.

**Figure 1:**
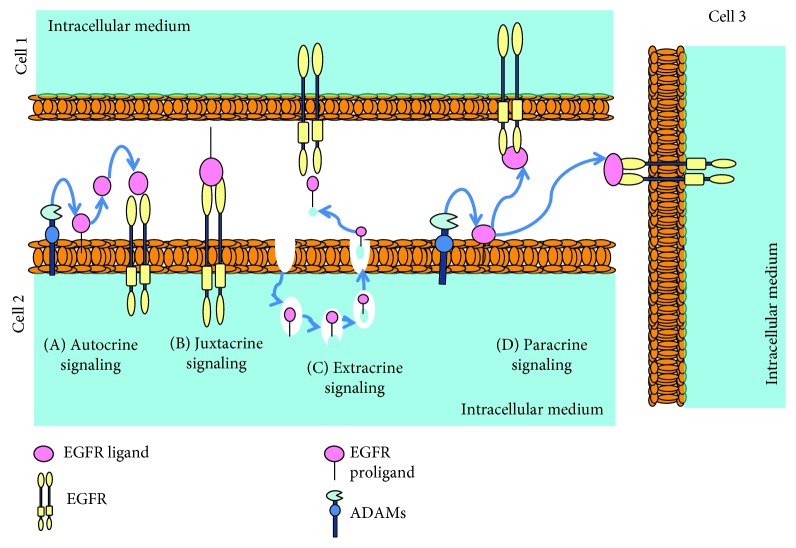
Types of signaling via epidermal growth factor receptor (EGFR) ligands: (A) the autocrine form if EGFR activation occurs in the same cell; (B) the juxtacrine form, when the ligand is anchored to the cell membrane; (C) the extracrine form, which combines features of autocrine, paracrine, and juxtacrine signaling as well as possibly endocrine signaling; and (D) the paracrine form if acting in a neighbouring cell. Adapted from Singh et al. 2016.

The first step of direct EGFR activation begins with the binding of specific ligands to the receptor. The seven official EGFR ligands have been extensively studied and share a common structure involved in EGF binding [17, 18], but information about the novel described ligands, such as CTGF, is scarce. EGFR ligands activate this pathway in different ways: (1) direct activation by soluble ligands, (2) the juxtacrine mode, when the ligand is anchored to the cell membrane, (3) autocrine signaling, in which EGFR activation occurs in the same cell, (4) the paracrine form if acting on a neighbouring cell [19], and (5) the extracrine form, which combines features of autocrine, paracrine, and juxtacrine signaling as well as possibly endocrine signaling, since EGFR and AREG can be detected in human plasma exosomes [20] (Figure 1).

All EGFR ligands can be found as soluble proteins, but some of them are also present as biologically active precursors anchored to the plasma membrane, including HBEGF, TGFA, AR, and BTC. The release of EGFR ligands from the cellular membrane is an important point in the EGFR transactivation process [21–25]. Interestingly, EGFR transactivation can be prompted by physiological and nonphysiological stimuli. The physiological stimuli capable of bringing about this effect include chemokines, adhesion molecules, and growth factors that require previous interaction with its specific receptors (GPCRs or not). EGFR transactivation by nonphysiological processes such as hyperosmolarity, oxidative stress, mechanical stress, ultraviolet light, and *γ* radiation is mediated by the inactivation of certain phosphatases that antagonize the intrinsic kinase activity of the receptor, thus allowing EGFR autophosphorylation [26].

The affinity of EGFR for its ligands depends on the tissue and pathological condition. Most of the studies have been done comparing the seven official EGFR ligands [17, 18]. These ligands display different ligand-biding affinities at around 3 orders of magnitude [17, 18]. Moreover, depending on the specific ligand that binds to EGFR, different cellular responses can be activated. Structural studies have described how EGFR activation occurs but ligand-related activation is less understood [18]. After EGFR ligand interaction, the receptor undergoes a conformational change leading to the formation of homo- or heterodimers. Then, the intracellular domain is activated in its tyrosine residues by phosphorylation, promoting the autophosphorylation of these same residues in their homologue. Phosphorylated residues in turn serve as a binding site for certain molecules that have domains of SRC homology; this interaction leads to different signaling cascades [27]. Earlier studies described that the different intracellular signaling triggered after EGFR activation depends on the phosphorylation of certain residues in the intracellular domain of the receptor. In SAA cells (NIH3T3 fibroblasts that overexpress human EGFR) stimulated with EGF, treatment with a phosphopeptide that blocks the autophosphorylation site Tyr1068 of EGFR induced a significant inhibition of EGFR/Grb2 interaction and RAS/MAP kinase activation [28]. EGFR activation translates signals to the nucleus, modulating the activity of transcription factors such as c-JUN, c-FOS, c-MYC, and NFKB and regulating gene transcription [16]. In bronchial epithelium, EGFR activation is linked to phosphorylation of the tyrosine residue (Tyr1173) associated with the activation of JNK, AP1, p38 MAPK, and NFKB pathways [29]. On the other hand, SRC kinase protein can act as cotransducer of EGFR signals and SRC-dependent EGFR activation mediated by EGFR phosphorylation of Tyr 845 and Tyr 1101 [30, 31].

**Figure 2:**
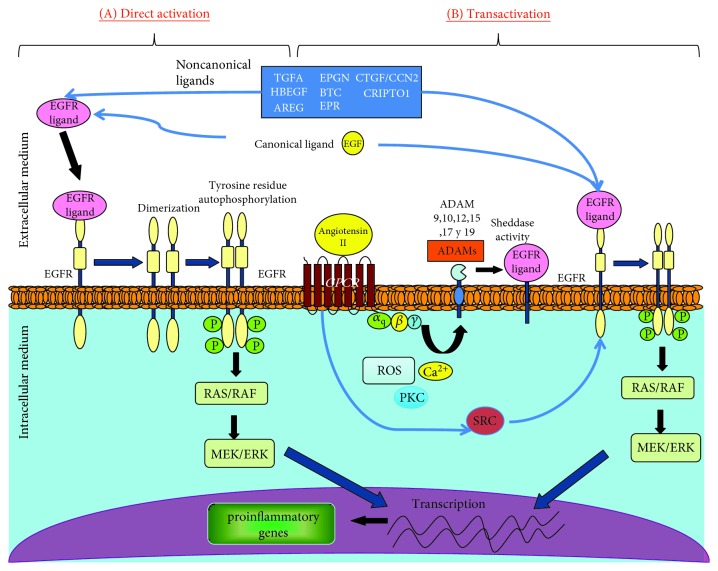
Different EGFR signaling systems in the inflammatory process: (A) direct ligand-receptor activation: the first step for the direct activation of EGFR begins with the binding of the ligand to the receptor. In general, EGFR ligands are located as inactive transmembrane precursors, which, in order to bind to their receptor, need to undergo proteolytic processing and be released as soluble ligands into the extracellular medium. This proteolytic processing is carried out by metalloproteases/disintegrins of the ADAM family. (B) Indirect ligand-receptor activation/transactivation: this process is triggered by the binding of molecules such as Ang II, thrombin, and ET1 to their specific receptor. After this binding, the release of second messengers is induced, such as intracellular Ca2^+^, ROS, and certain protein kinases such as PKC, which induces activation of metalloproteases/disintegrins of the family of ADAMs. After EGFR ligand interaction, the receptor undergoes a conformational change inducing the formation of homo- or heterodimers. Then, the intracellular domain is activated in its tyrosine residues by phosphorylation, promoting the autophosphorylation of these same residues in their homologue. Phosphorylated residues in turn serve as a binding site for certain intracellular kinases that are capable of activating EGFR independently to MMPs, as in the case of the SRC kinase.

EGFR transactivation (indirect activation) is triggered when different molecules bind to their specific receptors. A variety of these molecules have been identified and can be broadly categorized into the following groups: G protein-coupled receptors (e.g., Ang II and ET-1), cytokines (e.g., TNFA, TWEAK, and other TNF receptor family proteins), growth factors (e.g., TGFB and other proteins, including TPA and LPA), integrins, ion channels, and other physical stimuli [32–38]. Many of these molecules that transactivate EGFR are very relevant in renal damage, including Ang II, TGFB, and TNF receptor family proteins, showing the importance of EGFR transactivation in renal pathology. After the specific binding of these molecules to their own receptors, several second messengers, including intracellular Ca^2+^, reactive oxygen species (ROS), and protein kinases, such as PKC, can be released [39–41]. These intracellular signals trigger a signaling cascade, leading to the activation of metalloproteases/disintegrins from the family of ADAMs. In general, the EGFR ligands involved in EGFR transactivation are inactive transmembrane precursors located in the cellular membrane which need to undergo proteolytic processing and be released as soluble ligands into the extracellular medium in order to bind to EGFR [42], being the most studied EGF, TGFA, and HBEGF. This proteolytic processing is carried out by ADAMs. Therefore, EGFR transactivation via ADAMs leads to the release of EGFR from the cellular membrane and subsequent binding to EGFR and pathway activation. Alternatively, in some cases, EGFR transactivation can occur independently of MMPs/ADAMs and is mediated by intracellular protein kinases, as in the case of Src kinase [43–46] (Figure 2).

### 1.2. ADAMs: Key Proteins in EGFR Transactivation

ADAMs are a family of 23 glycoproteins expressed as transmembrane surface proteins consisting of an extracellular metalloproteinase domain followed by a disintegrin-like domain, a cysteine-rich domain, a transmembrane domain, and finally a cytoplasmic tail. Their proteolytic activity is mediated by the zinc-dependent metalloproteinase domain, with the other domains contributing to substrate recognition and regulation. These domains give them its characteristics of adhesion molecules and proteases [47]. The first ADAMs described were involved in reproductive functions, mainly spermatogenesis and the attachment of sperm to the ovule (ADAM1 and ADAM2). However, only 12 of the human ADAMs (ADAM8, 9, 10, 12, 15, 17, 19, 20, 21, 28, 30, and 33) contain a functional catalytic consensus sequence (HEXGEHXXGXXH). The physiological function of the proteinase-inactive ADAMs (ADAM2, 7, 11, 18, 22, 23, 29, and 32) remains largely unknown, although some members of this group play important roles in development and function as adhesion molecules rather than proteinases [48, 49]. Numerous transmembrane proteins have been identified as targets of ADAM-mediated proteolysis [50]. Some of these substrates can be cleaved by different ADAMs, while others appear to be specific to an individual ADAM.

Recently, the roles of different ADAMs such as ADAM9, 10, 12, 15, 17, and 19 in the release and/or activation of cell surface proteins have been described. Among those proteins cleaved by ADAMs are several EGFR ligands, such as EGF, TGFA, and HBEGF [47, 51]. Depending on the tissue, different ADAMs may be involved in the release of the EGFR ligands. For example, kidney angiotensin II- (Ang II-) induced transactivation of EGFR is mediated by ADAM17 and by the release of TGFA [52], while in the heart, ADAM12 regulates this process through the release of HBEGF [53].

These glycoproteins are synthesized in the Golgi apparatus; under the action of the furin protease, they undergo a conformational change that induces activation. In their active form, they are transported to the plasma membrane where they exert their sheddase activity on the inactive precursors of the EGFR ligands. Functional upregulation of ADAM activity is generally observed in association with cytosolic Ca^2+^ elevation, purinergic receptor agonists, or membrane-perturbating agents. In some cases, such as ADAM17, sheddase activity is amplified by other signaling pathways, including activation of protein kinase C (PKC) and receptor tyrosine kinases. Furthermore, certain physicochemical properties of the lipid bilayer also govern the action of ADAM proteases [54].

Once these precursors are proteolytically processed, they are released into the extracellular environment and can interact with EGFR and activate this signaling pathway [42]. Therefore, modulation of ADAM activity is of paramount importance to EGFR activation. Indeed, several strategies for ADAM inhibition are being considered as pharmacological targets. Thus, therapeutic strategies have focused on the inhibition, induction, and activation of ADAM expression as well as the use of small molecules blocking the active site and events blocking the domain for substrate recognition [55].

### 1.3. EGFR in Pathological Processes

Malfunctioning of EGFR, or its related pathways, has been shown to be relevant to the pathogenesis of several malignancies, playing a role in the development of ovarian, breast, and colorectal cancers as well as non-small cell lung and head and neck carcinomas [56]. Moreover, a strong correlation between EGFR expression and prognosis has been found in ovarian, head and neck, bladder, and esophageal cancers [57]. Thus, anti-EGFR therapy could have a place in the treatment of some of these tumors; in fact, the use of erlotinib or cetuximab may be employed as second-line therapy after failure of mono- or polychemotherapy in squamous cell lung carcinoma [58]. Recent studies suggest that EGFR activation can be involved in inflammatory diseases. Overexpression of EGFR may be related to a number of skin disorders, such as psoriasis or atopic dermatitis [59]. Apart from these spontaneously occurring diseases, it has been described that inhibition of EGFR by either monoclonal antibodies or small-molecule EGFR tyrosine kinase inhibitors brings about a monomorphic acneiform reaction, which, interestingly, is directly correlated with overall survival [60]. EGFR blockade is being explored as a new treatment for the disease [61], and future studies on inflammation are needed.

### 1.4. EGFR in the Kidney in Normal and Pathological Conditions

EGFR is expressed in the kidney, specifically localized in the glomerulus and the tubulointerstitial compartment [62]. This receptor plays a key role in renal electrolyte homeostasis [42]. However, its role in renal pathology is somewhat contradictory since both beneficial and deleterious actions have been found. Interestingly, upregulation of the EGFR pathway (including several of its ligands, as TGFA, HBEGF, and CTGF) has been described in human and experimental chronic renal pathologies, including glomerulonephritis, diabetic nephropathy, transplant rejection, and polycystic kidney disease [63–65], as well as in experimental models of acute kidney injury (AKI), such as ischemia/reperfusion or folic acid administration [66–68]. Intensive research in many experimental studies has shown that EGFR blockade exerts beneficial effects in progressive kidney disease, mainly ameliorating fibrosis [69, 70]. However, EGFR inhibition in AKI models exerts opposite results, presenting deleterious effects. Studies in models of ischemic injury using waved-2 mice (an EGFR mutation that induces a reduction in receptor tyrosine kinase activity) and wild-type mice treated with the EGFR kinase inhibitor erlotinib showed significantly decreased renal function in the depleted/treated mice [71, 72]. On the contrary, activation of the EGFR pathway can accelerate renal recovery in the early AKI phase by means of a mechanism that involves induction of renal tubular cell regeneration and protection of these cells from apoptosis, as demonstrated by the *in vivo* administration of several ligands (EGF, HBEGF) in experimental acute ischemic injury [69, 73–78]. These data were confirmed *in vitro* in cultured proximal tubular epithelial cells, in which activation of EGFR by the ligands EGF [76], HBEGF [46], and EPR [78, 79] induced cell proliferation and migration, supporting their protective role in renal repair after AKI. Thus far, there are no studies of CTGF modulation in experimental AKI.

Interestingly, the consequences of EGF signaling activation depend on species. In models of hydronephrosis, EGF administration causes cell death in mice, while it induces cell survival in rats [80]. Moreover, in a rat model of cisplatin-induced nephrotoxicity, the EGFR inhibitor erlotinib induced renoprotective properties by modulation of apoptosis and proliferation of tubular cells [81].

### 1.5. Official and Novel Described EGFR Ligands and Signaling in Renal Physiopathology

Most EGFR ligands have a similar globular structure, with a common fold defined by six conserved cysteine residues that form three disulphide bonds, termed EGF motifs, through which they interact with EGFR [82–86]. In contrast, CTGF does not possess an EGF motif and interacts with EGFR through its C-terminal domain [7] and future studies are needed to compare its structural binding to EGFR with other ligands. Several EGFR ligands contain amino-terminal heparin-binding domains (HBEGF domains), including HBEGF, AR, and CTGF [87, 88]. Contradictory results have been also reported in diabetic nephropathy. For instance, high glucose induces transactivation of EGFR and profibrotic responses in mesangial cells by increasing HBEGF release [89, 90]. However, Dey et al. [91] reported a beneficial effect of ADAM-mediated EGFR transactivation by bradykinin, leading to a decrease of podocyte permeability. EGFR ligands have generally been classified based on their interaction with EGFR and can be categorized into two classes: high affinity or low affinity. The high-affinity ligands are EGF, TGFA, HBEGF, and BTC, which bind to EGFR and have a dissociation constant (Kd) of between 1 and 100 nM, while the low-affinity ligands, AR, EPR, and EPGN, have a Kd greater than 100 nM [17]. CTGF has a Kd of 126 nM, which places it within the group of ligands of lower affinity [7].

### 1.6. EGF (Canonical Ligand)

EGF is the canonical EGFR ligand and the one with the highest EGFR affinity described to date. EGF was identified from submaxillary gland extracts during nerve growth factor studies [92]. The kidney is the key source for EGF production, and several studies have identified elevated urine EGF levels as an independent risk factor for CKD progression [93–97].

EGF is produced in the kidney as a membrane-anchored prepro-EGF located in the apical membrane of epithelial cells [93, 98, 99]. Earlier studies conducted in rats showed positive EGF immunostaining, mostly in the proximal tubules, as well as increased *EGF* mRNA levels in the thick ascending limb of Henle's loop and distal convoluted tubules [100, 101]. EGF regulates physiological processes in the kidney. Several studies have shown the important role of EGF on sodium and magnesium transport through the regulation of epithelial ion channels such as epithelial sodium channel (ENaC) or Na^+^/K^+^/2Cl^−^ cotransporter (NKCC1), among others [102–105]. Many studies have described the importance of EGF in polycystic kidney diseases [106–108] as well as the role of EGF in cell proliferation, including in tubular epithelial cells [109, 110]. Exogenous EGF administration has been found to reduce tissue injury caused by ischemia/reperfusion injury (IRI) showing beneficial antiapoptotic and antioxidant effects [111–113]. However, *in vitro* studies described prooxidative effects. In mammalian cells, EGF enhances the production of intracellular reactive oxygen species by dual oxidase 1 [114] and these oxidative species, which are induced by EGF, modulate ADAMs (positive regulator of EGFR signaling) or protein tyrosine phosphatases (negative regulator of EGFR signaling) [115–118]. AKI is a multifactorial pathology characterized by renal tubular damage, inflammation, and, frequently, a transient decrease in renal function. After the initial injury, there is a recovery phase, associated with proximal tubular cell proliferation and migration (as part of a regeneration process). But if this regenerative process fails to resolve, it may lead to fibrosis and loss of renal function [42, 119, 120]. Several experimental studies have shown the involvement of EGF in renal regeneration after IRI damage [121–123], but studies on EGF signaling in renal inflammation are scarce.

### 1.7. Transforming Growth Factor-*α* (TGFA)

TGFA is one of the most widely studied EGFR ligands. TGFA is expressed in normal adult human kidney [121], and TGFA protein is detected in the urine of healthy human subjects [122]. Several experimental models of renal damage have described upregulation of TGFA. In an experimental model of renal mass reduction, elevated TGFA protein levels were markedly increased after nephron reduction and prior to the development of renal lesions [123]. Experimental studies have demonstrated the involvement of TGFA in renal fibrosis, as in the model of Ang II-induced renal damage [52]. Moreover, elevated mRNA expression levels of *TGFA* were found in inflammation-induced renal damage triggered by systemic administration of TWEAK. In addition, treatment with a pharmacological inhibitor of ADAM17 (WTACE2) diminished renal inflammation associated with downregulation of TGFA and inhibition of EGFR pathway activation [38]. In *in vitro* studies in cultured tubular epithelial cells, stimulation with recombinant TGFA upregulated proinflammatory gene expression [38]. In these cells, upregulation of proinflammatory cytokines and chemokines induced by stimulation with aldosterone and TWEAK was blocked by a TGFA-neutralizing antibody [124]. Additionally, in Xenopus 2F3 cells, chronic treatment with TGFA over 24 hours inhibits the epithelial sodium channel ENaC by decreasing the number of channels in the membrane through the modulation of MAPK1/2 pathways but acute treatment with TGFA for 1 hour activates ENaC via PI-3 kinase [125]. In models of middle cerebral artery occlusion in mice and rats, TGFA treatment significantly reduced infarct size, suggesting that TGFA can induce angiogenesis, neurogenesis, and neuroprotection after stroke [126, 127].

Several studies have investigated the effect of TGFA loss on different pathological conditions. Research using a model of acute intestinal mucositis in *TGFA* knockout mice described that the lack of TGFA in intestinal epithelial cells resulted in higher apoptosis and lower proliferation [128]. In a model of bleomycin-induced lung injury, *TGFA*-null mice exhibited diminished pulmonary inflammation and fibrosis compared to wild-type mice [129]. Another study about peripheral nerve injury showed that *TGFA* knockout mice have no effect in regeneration process, probably due to compensatory expression mechanisms of other EGFR ligands [130]. These studies demonstrated that EGF-related growth factors could present specific and unique functions in certain tissues and cells.

### 1.8. Heparin-Binding Epidermal Growth Factor (HBEGF)

HB-EGF is a 22 kDa protein originally identified in macrophage-like U-937 cells [131]. HBEGF is synthesized as a transmembrane precursor protein (pro-HBEGF) that can be cleaved by metalloproteinases such as ADAM17 to release a mature soluble HB-EGF (sHBEGF), a different form that is more functionally active *in vivo* than the precursor protein [82, 132]. The soluble form of HBEGF is capable of linking to heparan sulfate proteoglycans present in the cell surface, favouring local expression and accumulation of growth factors [133]. HBEGF participates in several physiological and pathological events, including wound healing [134], atherosclerosis [135], and tumor progression [136].

Some studies have analysed the role of HBEGF in renal pathology. Studies carried out on *HBEGF*-deficient mice showed the involvement of this ligand in podocyte damage in progressive glomerulonephritis. The loss of *HBEGF* was associated with lower renal inflammatory infiltration and decreased albuminuria levels prior to the appearance of renal cell proliferation [137]. Several studies using mice with specific *HBEGF* deletion in endothelium demonstrated attenuated renal damage in streptozotocin- (STZ-) induced diabetic renal injury [138] and in response to Ang II infusion [139]. In these conditional knockout mice, the inflammation in the perivascular area or renal interstitium (tested by F4/80- and CD3-positive stained cells) and the renal fibrosis caused by Ang II were significantly reduced compared to those in control mice [139]. Pharmacological blockade of ADAM17 by WTACE2 in a model of renal injury induced by TWEAK administration reduced *HBEGF* renal mRNA expression levels associated with lower inflammatory cell infiltration [38]. *In vitro* studies have clearly demonstrated that HBEGF regulates cell proliferation, including in glomerular epithelial cells [69, 140], and also regulates proinflammatory gene expression, as observed in cultured tubular epithelial cells [38]. In inner medullary collecting duct cells, the sustained exposure to sHBEGF induces the transition from an epithelial to a mesenchymal phenotype by upregulating the E-cadherin transcriptional repressor SNAIL2, thus contributing to renal fibrosis [141].

### 1.9. Amphiregulin (AREG)

AREG is constitutively expressed in different cell types during development and homeostasis [142] and participates in several physiological processes, including the regulation of pulmonary morphogenesis [143] and the proliferation of keratinocytes [144]. Although AREG was originally described as an epithelial cell-derived factor, multiple studies have shown that it can also be expressed by activated immune cells in different inflammatory processes. Emerging evidence shows that AREG plays a critical role in restoring tissue integrity after infection or inflammation [145–148] and induces tolerance by promoting the restoration of tissue integrity after damage associated with acute or chronic inflammation [149, 150]. The immune system plays an important role in the EGFR signaling pathway, thus contributing to the progression of the inflammatory process. Cells such as basophils express high amounts of AREG after exposure to IL3 [151] and other types of immune cells such as neutrophils, CD8 T cells, and T regulatory lymphocytes [149]. Interestingly, AREG is expressed only in proinflammatory-type M1 macrophages [152]. Inflammation, ischemia, and hypoxia induce AREG expression in the brain. Under these situations, glial cells show upregulation of AREG, which protects against neuronal cell death. In neuro-2a cells, administration of AREG inhibits endoplasmic reticulum stress and cell death [153].

There are few studies about the role of AREG in kidney damage. In autosomal dominant polycystic kidney disease, the use of anti-AREG antibodies and inhibitors of activator protein-1 (AP1) can reduce cell proliferation in cystic cells by reducing AREG expression and EGFR activity [154]. In a model of streptozotocin-induced diabetes, the genetic blockade of *EGFR* or pharmacological inhibition using erlotinib showed a downregulation of phospho-AKT, CTGF, and AREG expression compared to that in diabetic mice [155]. However, there are no studies assessing the role of AREG gene deletion of direct modulation in experimental renal disease.

### 1.10. Betacellulin (BTC)

BTC was first described in 1993 by Shing and et al. as a mytogen from pancreatic B cell tumors [5]. BTC was detected in the normal kidney at low levels; its location in the nephron remains unclear [156]. BTC could have different roles depending on the organ where it is expressed. A murine model of *BTC* overexpression demonstrated a reduction in body weight in *BTC* transgenic animals, which was accompanied by a reduction in kidney and pancreas weight, whereas the lungs of these animals were overgrown and their hearts had the same weight as those in controls [157]. Regarding inflammation, few studies have evaluated the role of BTC in this process, and to our knowledge, there is none in the kidney. One study demonstrated that BTC upregulates COX-2 expression in human granulose cells [158]. With regard to particular diseases, elevated BTC levels have been described in rheumatoid arthritis patients [159] and other authors observed that *BTC* overexpression protects against acute pancreatitis by activating stress-activated protein kinase [160].

### 1.11. Epiregulin (EPR)

EPR was originally purified from conditioned medium of the fibroblast-derived tumor cell line NIH3T3/T7 [6] and is another example of the less widely studied EGF ligands. Higher EPR concentrations were found in patients with inflammatory diseases [161], including patients suffering from rheumatoid arthritis [159]. In this pathology, EPR inhibition suppresses the development of experimental autoimmune arthritis [161]. EPR regulates several immune-related processes. EPR is involved in peptidoglycan-mediated proinflammatory cytokine production in antigen-presenting cells and in innate immunity [162]. In a model of wound healing in corneal epithelial cells, *EPR* knockout mice presented an increased number of infiltrating cells in the wound area and this difference was related to the upregulation of several proinflammatory factors, including IL6, CXCL1, CXCL2, and CCL2 [163]. In the kidney, EPR promotes proliferation and migration of renal proximal tubular cells [78]. In 2016, Boyles et al. [164] commented on unpublished data with regard to the potential beneficial effects of a specific EPR neutralization antibody in experimental diabetes. However, the direct role of EPR on kidney inflammation requires further study.

### 1.12. Epigen (EPGN)

EPGN was first identified in 2001 by Lorna Strachan. It consists of 152 amino acids and a transmembrane domain. EPGN is present in many tissues such as the testes, heart, and liver and is characterized as a low-affinity ligand [165, 166]. Several studies have shown that EPGN participates in cell proliferation. EPGN is a mitogen for HaCaT cells [165]. In epithelial cells, EPGN stimulates phosphorylation of c-ERBB1 and MAP kinase proteins [165]. The EPGN transgenic overexpression during embryonic development induces sebaceous gland hyperplasia [167], and the activation of NRF2 causes sebaceous gland enlargement in an EPGN-dependent manner [168]. *EPGN*-null mice exhibit peripheral demyelinating neuropathy that induces muscular dystrophy [169]. Data on inflammation, however, is scarce. One study described the involvement of EPGN in the inflammatory process in the skin via ERK pathway activation [170]. Other studies reported that recombinant EPGN is unable to activate ERBB2 in the presence of other ERBBs. Additionally, soluble EPGN has more mitogenic activity than EGF, although its binding affinity is lower [171].

### 1.13. The Two Unofficial/Novel Ligands

#### 1.13.1. Teratocarcinoma-Derived Growth Factor 1 (TDGF1; CRIPTO1; CR-1)

CRIPTO1 is another molecule that binds to EGFR but differs to official EGFR ligands because it does not possess an EGF-like motif. In addition to binding to EGFR, CRIPTO1 also acts as a coreceptor for the TGFB subfamily. CRIPTO1 is critically important in early embryogenesis, maintenance of stem cells, and the progression of some types of cancer [172]. There are few studies on the role of this EGFR ligand in pathophysiological processes. A cancer study reported that CRIPTO1 was expressed in a certain type of non-small cell lung cancer that causes intrinsic resistance to specific inhibitors of EGFR tyrosine kinase activity and participates in EMT [173]. A recent study suggests a potential role of this EGFR ligand in cardiac repair. In human cardiac ventricular fibroblasts, CRIPTO1 production was increased in response to reparative factors, such as NRG1B, and the blockade of PI3K, ERBB2, and ERBB3 by pharmacological inhibitors or neutralizing antibodies significantly reduced CRIPTO1 levels [174]. Currently there are no studies about the role of CRIPTO1 in inflammatory processes or in kidney diseases.

#### 1.13.2. Connective Tissue Growth Factor (CCN2/CTGF): A Newly Described EGFR Ligand

CTGF (also known as CCN2) is a cysteine-enriched secretable matricellular protein with a molecular weight of 38 kDa. CTGF was identified in the conditioned medium of endothelial cells of the umbilical cord vein [175]. This protein has a modular structure made up of a secretory peptide at the N-terminal end followed by 4 functional modules [176]: (1) the insulin-like growth factor- (IGF-) binding domain, which stimulates the production of matrix proteins in renal cells [177–179]; (2) the von Willebrand factor type C domain, which is rich in cysteines and participates in protein oligomerization and synthesis. In Xenopus cells, CTGF binds directly to TGFB through this domain and promotes binding to its receptor, leading to the activation of the Smad response promoter [180]; (3) the thrombospondin-1 domain, which is involved in the union of soluble macromolecules or matrix proteins and participates in the union of CTGF to VEGF [181, 182]; and (4) the C-terminal domain, a dimerization domain involved in binding to the cell surface, possesses mitogenic activity for fibroblasts, and is responsible for the interaction with fibronectin [183]. This domain contains heparin-binding EGF sites [87, 88] and a region with a cysteine knot motif that resembles PDGF, TGFB, and NGF [184]. Finally, the N-terminal domain contains putative binding sites for IGF and TGFB.

Between module 2 and module 3, there is a hinge region that can be processed by multiple proteases, including the MMPs 1, 2, 3, 7, 9, and 13, generating two protein portions (one with the N-terminal domain and the other with the C-terminal domain), both with biological activity. This region can also be proteolyzed by elastase and plasmin, which can cleave the individual modules to produce four fragments [185]. In addition, it has been observed *in vitro* that MMP2 processes CTGF, thereby generating the C-terminal fragment of 10–12 kDa. In biological fluids and in the medium of cells in culture, the presence of CTGF has been described in its different forms: complete molecule CTGF, C-terminal fragment, and the N-terminal fragment [186, 187]. However, the *in vivo* biological effects of CTGF and its fragments have not been investigated in depth.

### 1.14. Role of CTGF in Pathological Processes: A Key Mediator in Renal Inflammation

CTGF plays an important role in multiple cellular processes such as development, differentiation, cell proliferation, extracellular matrix (ECM) remodelling, and angiogenesis [8]. According to the cell type, a large variety of factors and molecules are involved in the induction and regulation of CTGF expression, including GPCR agonists, such as Ang II; growth factors such as TGFB, BMP, VEGF, IGF, GMCSF, and IL4; high concentrations of glucose; AGEs; hypoxia; mechanical stress; and oxidative stress [188–196].

CTGF is a developmental gene that is not expressed in adult tissues. However, under pathological conditions, CTGF could be induced in several diseases such as scleroderma, pulmonary fibrosis, and hepatic fibrosis [178, 196, 197] and in a multitude of renal diseases, including diabetic nephropathy [10, 198, 199]. In several independent studies, CTGF has been proposed as a biomarker for human diabetic nephropathy and other forms of CKD [8, 10–12] and also for cardiac dysfunction in patients exhibiting myocardial fibrosis and chronic heart failure [13]. Plasma CTGF levels predict end-stage renal disease and mortality in diabetic nephropathy [12]. Moreover, urine CTGF can also be used as predictor/biomarker of CKD, including diabetic nephropathy [9, 10, 14]. Patients with reduced right ventricular function had higher plasma CTGF levels than those with normal or mildly reduced right ventricular function [15].

Initial studies demonstrated the role of CTGF as a mediator of the profibrotic action of TGFB [200] and other factors involved in renal damage, such as Ang II [201] or endothelin-1 [202]. Additionally, experimental studies showed that blockade of endogenous CTGF using different approaches, including antisense oligonucleotides or gene silencing, demonstrated beneficial effects in fibrotic-related diseases, including experimental lung, liver, and vascular damage, as well as models of chronic renal damage, including diabetic nephropathy [200, 201, 203–205]. Interestingly, CTGF exerts opposite effects in other pathologies in much the same way as other EGFR ligands. CTGF overexpression conferred cardioprotection in Ang II-infused mice and in ischemia-reperfusion injury [206, 207]. However, the role of CTGF in AKI has not been investigated in depth. A recent study by our group described the beneficial effects of *CTGF* gene deletion, reducing proliferation, the induction of the G2M phase of cellular cycle, and fibrosis in the kidney using a model of CTGF injection over 10 days [70]. Near total inhibition of *CTGF* below baseline levels reduced tubulointerstitial fibrosis in different models of renal damage, such as folic acid administration or obstructive nephropathy [70, 208].

Many reports suggest that CTGF can also be considered a cytokine involved in the regulation of immune and inflammatory responses. CTGF can activate several cells of the immune system. CTGF is a chemotactic factor for immune cells, including mononuclear cells [209], and induces cell adhesion and migration [210]. In human CD4 lymphocytes, CTGF, in combination with IL16, contributes to Th17 differentiation [211]. Interestingly, monocyte-derived macrophages do not produce CTGF on stimulation with TGFB, lipopolysaccharide, but CTGF is taken up by macrophages *in vitro* [209]. In an early study, our group demonstrated that *in vivo* administration of the C-terminal CTGF fragment induced an acute renal inflammatory response, characterized by infiltration of inflammatory cells in the kidney (lymphocytes and macrophages) and activation of the NFKB and subsequent induction of proinflammatory factors such as CCL2, CCL5, and IL6 [212]. In later studies, we found that CTGF induces a sustained renal inflammatory response linked to activation of the Th17 response, characterized by the presence of interstitial infiltration of Th17 (IL17A^+^/CD4^+^) cells and upregulation of Th17-related factors (STAT3 and ROR*γ*t) [211]. Recent evidence suggests that CTGF can also regulate inflammation in other pathological conditions. Studies performed with conditional *CTGF* knockout mice have found lower macrophage accumulation and downregulation of proinflammatory factors in peritoneal-induced damage [213]. In experimental models of alcohol-induced inflammatory process in the pancreas, overexpression of *CTGF* in mice plays a novel role, regulating inflammation by increasing infiltration of macrophages and neutrophils and increasing inflammatory mediators such as IL1B or CCL3 [214]. In another study in a model of skin fibrosis induced by Ang II, pharmacological blockade with a neutralizing antibody against CTGF mitigated the inflammation and fibrotic process in the dermis and diminished the number of cells expressing PDGFRB, procollagen, *α*SMA, pSMAD2, CD45, and FSP1 [215].

**Figure 3:**
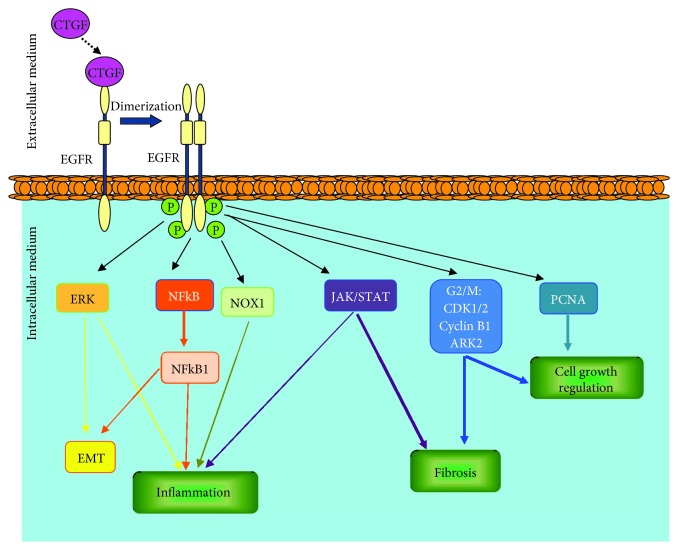
Different signaling pathways related to EGFR activation induced by CTGF/EGFR interaction. CTGF binds to EGFR through its C-terminal module. This interaction activates the EGFR signaling pathway linked to the modulation of different pathways closely related with cell growth, oxidative process, inflammation, EMT, and fibrosis in renal damage.

Recent studies have analysed the relationship between CTGF and other signaling pathways. Several have demonstrated the crucial role played by integrins, proteoglycans heparan sulfate, and low-density protein receptors in CTGF cellular responses [216]. The existence of CTGF binding sites in the cell membrane was suggested in 1998 in chondrocyte studies [217]. In 2005, a potential CTGF receptor was described in mesangial cells, the receptor tyrosine kinase of nerve growth factor (TRKA), a member of the TRK membrane receptor family (TRKA, TRKB, and TRKC) [218]. Some studies have confirmed that CTGF also activates TRKA in murine cardiomyocytes and tubuloepithelial cells [7, 219]. In 2013, we described that CTGF can bind to EGFR through its C-terminal module and via a process modulated by *α*V*β*3 integrin [7]. The CTGF-EGFR interaction activates this signaling pathway linked to the modulation of proinflammatory factors and the recruitment of lymphocytes and macrophages in the kidney [7]. At the vascular level, we also observed that CTGF-EGFR activation is linked to the oxidative process, endothelial dysfunction, and vascular inflammation through the NOX1 and NFKB pathways [220]. These results demonstrate that the activation of the EGFR pathway by the new ligand CTGF regulates inflammatory processes (Figure 3) and identify EGFR as a potential therapeutic target for the treatment of chronic kidney disease and vascular diseases closely linked with kidney damage.

In summary, EGFR activation has dual effects in AKI or CKD, ameliorating renal damage in experimental AKI by activating the regenerative process that occurs following acute renal damage through the induction of proliferation and migration of tubular cells. In contrast, EGFR activation exerted deleterious effects on CKD by activation of a fibrotic-related process, as observed in long-term models of renal damage [42, 70, 119, 120] (Figure 3).

### 1.15. Role of ERBB Crosstalks in Renal Inflammation

Previous studies described the possible crosstalk between EGFR and other receptors. The most obvious EGFR crosstalk is related to its heterodimerization with other members of the ERBB family (ERBB2, ERBB3, and ERBB4), whose function is amplifying and diversifying the signals [221–223].

Depending of the type of dimerization of ERBB1 (with itself or with other ERBB receptors as ERBB2), the signaling is very different [224, 225]. Only ERBB1/EGFR, ERBB3, and ERBB4 are able to bind ligands, an obligatory process for activation of the tyrosine kinase domain and intracellular signaling [226, 227]. ERBB2 has no known ligands, and it acts as a signal transducer in the recruitment of other components of the heteromeric complexes like ERBB1, ERBB3, or ERBB4 [228, 229]. ERBB3 has no kinase activity, and the biological relevance of the complex formation with other ERBB receptors (ERBB2-ERBB3 and ERBB2-ERBB4) has been analysed in studies with *ERBB2*,*3*,*4*-null mice [230–233].

As described above, EGFR transactivation can be induced by several GPCRs, different cytokines, integrins, and diverse tyrosine kinase receptors (TKRs) [36, 234, 235]. Moreover, it is possibly a crosstalk induced by ligand-independent EGFR transactivation, which consists of physical interactions between EGFR and other receptors such as platelet-derived growth factor receptor (PDGFR) [236] or IGF1R [237] and c-MET [238].

Several studies developed in monocytes have described a crosstalk between EGFR and GPCRs, linked to the regulation of inflammatory responses. The pharmacological blockade of EGFR or N-formyl-l-methionyl-l-leucyl-phenylalanine (fMLP) receptor, with AG1478 or cyclosporine H, respectively, decreased oxidative stress, CD11b upregulation, and EGFR/fMLP phosphorylation induced by their respective ligands. This crosstalk is SRC and ERK dependent [239]. In cervical cancer, GPCR TF-PAR2 (tissue factor protease-activated receptor 2) transactivates EGFR and mediates resistance to cisplatin, decreasing cisplatin-induced apoptosis [240]. Another study in monocytes has described a crosstalk between EGFR and TRKA in response to the stimulation of GPCRs [239], which further confirms the interaction between these two receptors. TRKA is a member of the TRK membrane receptor family (TRKA, TRKB, and TRKC). These receptors interact with neurotrophins and form homo/heterodimers with the low-affinity neurotrophin receptor, p75NTR [241].

There are few studies about EGFR crosstalks in renal inflammation. Most of them are related to EGFR transactivation. Lautrette et al. [52] demonstrated a possible crosstalk between EGFR and Ang II receptors in the kidney. In this in vivo study, the authors showed that Ang II is capable of transactivating EGFR and overexpression of a dominant negative isoform of EGFR prevents the frequency and severity of renal lesions in Ang II mice as well as interstitial cell infiltration.

In rat renal fibroblasts, palmitic acid (PA) activated the EGFR signaling pathway through TLR4/c-Src signaling. Previous studies described that fatty acids directly activate Toll-like receptor 4 (TLR4) and this process can induce c-SRC kinase activation [242]. In NRK-52E cells, the pharmacological blockade of EGFR or c-SRC previous to PA stimulation suppressed EGFR activation and its downstream signaling pathways ERK and AKT, closely related with renal inflammation and oxidative stress. The genetic silencing of *TLR4* through siRNA transfection blocked the PA-induced phosphorylation of EGFR, c-SRC, ERK, and AKT [243].

A study carried out in hepatocyte growth factor (HGF) transgenic mice demonstrated that the HGF/c-MET system significantly reduced LPS-induced renal and vascular injuries by the diminution of inflammation and ROS production, though EGFR ubiquitin degradation [244].

In vitro studies have described an EGFR crosstalk with aldosterone receptors. In cultured tubular epithelial cells, aldosterone caused EGFR transactivation, by a process mediated by ADAM17 and release via TGFB, and upregulated proinflammatory genes, via ERK and STAT1 activation [124]. In mesangial cells, aldosterone also activates EGFR linked to ROS production, ERK signaling, and modulation of cell growth [245].

A study developed in renal cells using gene silencing and pharmacological inhibitors of EGFR and TRKA demonstrated a clear crosstalk between EGFR and TRKA in response to stimulation with CTGF [7]. The analysis of the phospho-proteomic profiles of TRKA and EGFR shows a considerable similarity in the signaling originated by these RTKs [246]. EGFR crosstalk with TNF-related proteins is also involved in renal inflammation. TWEAK, a TNF member, is a cytokine that engages its receptor Fn14 to activate ADAM17, which releases the mature ligands HBEGF and TGFA that, in turn, transactivate EGFR. In cultured tubular epithelial cells, Fn14 gene silencing inhibited TWEAK-induced EGFR phosphorylation. Conversely, EGFR inhibition blocked TWEAK-induced responses, including activation of the ERK kinase pathway and upregulation expression of proinflammatory factors [38]. Moreover, pharmacological EGFR blockade inhibited TWEAK-induced renal inflammation. In vitro studies in renal cells have described that CTGF is involved in TGFB-induced EGFR transactivation [7]. However, the functional consequence of this EGFR/TRII crosstalk has not been investigated.

In conclusion, there are some possible crosstalks between ERBB1 and other receptors and their different interaction will trigger diverse signaling pathways, as well as amplify and diversify the intracellular signals related to inflammation and other key processes in kidney diseases. However, more studies in this topic are needed.

### 1.16. Therapy Targeting the ERBB1 Receptor in Kidney Disease

Several experimental studies have suggested that blocking EGFR could be an important tool to treat kidney diseases [42], especially by regulating inflammation, cell proliferation, and fibrosis [7, 38, 69]. Studies conducted in an autosomal recessive polycystic kidney model showed that treatment with an EGFR kinase inhibitor decreased the formation of cysts and improved renal function. In addition, similar results were observed using *WAVED-2* mutant mice (which present a point mutation in EGFR that reduces their 90% tyrosine kinase activity) [247]. In subsequent studies using the same mice model, it was observed that the beneficial effect of EGFR blockade was increased when is combined with the inhibition of ADAM17 [248]. Previous studies described that the EGFR blockade in different mice models of renal damage, induced by CCN2 and TWEAK injection, reduced the inflammatory infiltration of T lymphocytes and macrophages as well as the gene and protein expression of the proinflammatory mediators CCL2, CCL5, or IL6 [38]. In vitro studies developed in tubuloepithelial cells and an experimental model of renal inflammation in mice induced by TWEAK injection showed that the pharmacological blockade of ADAM17 with TAPI-2 and WTACE-2, respectively, inhibited the upregulation of proinflammatory mediators at gene and protein levels. Studies performed in models of renal mass reduction (subtotal nephrectomy) and prolonged ischemia showed that the truncated expression of a dominant negative of EGFR in proximal tubular cells decreased the infiltration of mononuclear cells, the accumulation of interstitial collagen, and renal tubular proliferation [249]. In diabetic rats, treatment with EGFR kinase inhibitors decreased the proliferation of tubuloepithelial cells, in addition to increase glomerular size [77]. In experimental models of hypertension induced by several factors (Ang II, leptin, monocrotaline, or ET1), EGFR blockade by different approaches including antisense oligonucleotides for EGFR, inhibitors of the EGFR kinase, and mutated *WAVED-2* mice reduced the characteristic effects of tissue damage observed in these models [250–253]. Systemic administration of Ang II induces severe fibrotic lesions in the kidney. However, the infusion of this peptide in mice that express a dominant negative form of the renal tubular-specific EGFR, protected them against the lesions produced by Ang II. In addition, it has been observed that in knockout mice for *TGFA* and in mice treated with a specific inhibitor of ADAM17, renal fibrosis induced by Ang II decreases [52].

The origin of the study of the EGFR signaling pathway was in tumor pathology [254, 255]. In various types of cancer, including tumors of the head, neck, lung, and breast and colorectal tumors, the ErbB family of receptors (EGFR/HER1/ERBB1, HER2/neu/ERBB2, HER3/ERBB3, and HER4/ERBB4) is unregulated, producing an inappropriate cell stimulation [254, 256]. In malignant tumors, it has been described that HER2 and EGFR/HER1 are overexpressed and it has been established that overexpression of EGFR correlates with a worse clinical prognosis [257]. In the specific case of lung cancer, EGFR overexpression has been described in 90% of tumors. Several mechanisms can trigger an aberrant expression of EGFR, including in particular the overexpression of the protein, its gene amplification, appearance of mutations, the overexpression of EGFR ligands, and finally the loss of the regulatory mechanisms of these processes [256]. It should be noted that mutations in this receptor are one of the indicators that correlate best with the efficacy of EGFR inhibitors [256]. Specifically, a marked beneficial effect and greater response to treatment with these EGFR inhibitors have been observed in patients with mutations in exon 19 (codons 746–750) and exon 21 (substitution of leucine by arginine in codon 858 (L858R)) of EGFR compared to patients without these mutations [258].

The EGFR signaling pathway plays a crucial role in tumor processes since it modulates the cell cycle, inhibits apoptosis, induces angiogenesis, and promotes the motility of cancer cells and metastasis [259]. The first therapeutic approaches against EGFR began with the development of reversible pharmacological inhibitors of EGFR such as gefitinib (competes with ATP to bind the intracellular tyrosine kinase domain of EGFR, preventing its phosphorylation). Subsequently, erlotinib was designed, which presented a better pharmacokinetic and toxicity profile [260]. However, the response to gefitinib and erlotinib did not improve survival and improvements were only observed in those patients with mutations in EGFR [261, 262]. Another newly developed inhibitor is afatinib, with dual specificity against EGFR/ERBB1 and HER2/ERBB2 [263]. Due to the involvement of these pathways in embryonic development and cell proliferation, most efforts so far are focused on anti-EGFR therapies. These therapies involve the use of tyrosine kinase inhibitors, which are small molecules that bind intracellularly and interfere with signaling of the receptor, and the use of monoclonal antibodies that block the extracellular domain of the EGFR kinase. Among the developed antibodies, cetuximab stands out, which improved the survival rates in patients with lung cancer and colorectal cancer in combination with chemotherapy [264, 265]. In a kidney cancer study, administration of a murine/human chimeric anti-EGFR antibody (C225) was shown to inhibit the growth of normal renal cell carcinoma explants in NUDE mice [266].

EGFR regulates vascular homeostasis and pathophysiology. Studies with spontaneously hypertensive rats showed that vascular smooth muscle cells expressed high levels of EGFR and increased proliferation [267]. In models of experimental hypertension, EGFR blockers reduced blood pressure elevation and improved vascular lesions [69, 250, 268–270]. In atherosclerosis, increased expression of EGFR and some of its ligands, such as HBEGF, were described in the different stages of the atherogenic process [135, 271–273]. EGFR activation has also been involved in the vascular complications of diabetes [274–276].

### 1.17. Future Perspectives

All these studies show the complexity of EGFR pathway activation and its involvement in the pathogenesis of kidney damage. The canonical EGFR ligand EGF participates in acute renal damage mainly regulating cell proliferation, and future studies focusing on its role in renal regeneration are important. The other official ligands, TGFA and HBEGF, have an important role in the process of EGFR transactivation, by modulating key factors of renal damage, including Ang II, aldosterone, and TWEAK, mainly by regulation of renal inflammation. The recently described EGFR ligand CTGF is a potential therapeutic target that exerts proinflammatory and fibrotic properties, although more research is needed to completely understand EGFR binding and its involvement in EGFR transactivation in vivo. These data suggest that inhibiting EGFR or some of its ligands is an interesting therapeutic strategy for CKD and future studies are warranted.

## Abbreviations

AREG: Amphiregulin
EPGN: Epigen
aa: Amino acids
ADAMs: Membrane-anchored disintegrin metalloproteases
ADPKD: Autosomal dominant polycystic kidney disease
AKI: Acute kidney injury
AKT: Protein kinase a
Ang II: Angiotensin II
AP1: Activator protein 1
BMP: Bone morphogenetic protein
BTC: Betacellulin
CCL3: Chemokine (C-C motif) ligand 3
CCN2/CTGF: Connective tissue growth factor
CKD: Chronic kidney disease
COX2: Cyclooxygenase 2
CYR61: Protein rich in cysteines 61
EGF: Epidermal growth factor
EGFR/c-ERBB1: Epidermal growth factor receptor
EMT: Epithelial mesenchymal transition
EPR: Epiregulin
ERK: Intracellular signal regulation kinase
ET1: Endothelin-1
FGF: Fibroblast growth factor
GMCSF: Granulocyte macrophage colony-stimulating factor
GPCRs: Receptors coupled to G proteins
HBEGF: Heparin-binding epidermal growth factor
HER2/neu (ERBB2): Receptor tyrosine-protein kinase erbB-2
HER3 (ERBB3): Receptor tyrosine-protein kinase erbB-3
HER4 (ERBB4): Receptor tyrosine-protein kinase erbB-4
HGF: Hepatocyte growth factor
IGF: Insulin-like growth factor
IL1B: Interleukin 1 beta
IL4: Interleukin 4
IL6: Interleukin 6
IRI: Ischemia/reperfusion injury
JNK: c-Jun N-terminal kinase
LPA: Lysophosphatidic acid
LPR: Low-density protein
MAPK: Protein kinases activated by mitogen
MCP1/CCL2: Monocyte chemoattractant protein-1
MMPs: Metalloproteinases
NFKB: Nuclear factor-κB
NGF: Nerve growth factor
NKCC1: Na^+^/K^+^/2Cl^−^ cotransporter
PDGF: Platelet-derived growth factor
PDGFRB: Platelet-derived growth factor receptor beta
PA: Palmitic acid
PI3K: Phosphatidylinositol 3-kinase
PKC: Protein kinase C
PLC: Phospholipase C
RANTES/CCL5: Regulated on activation, normal T expressed, and secreted
ROR*γ*t: RAR-related orphan receptor gamma
ROS: Reactive oxygen species
RTKs: Receptors with tyrosine kinase activity
SMAD: Mothers against decapentaplegic homolog
SRC: Tyrosine-protein kinase
STAT3: Signal transducer and activator of transcription 3
STZ: Streptozotocin
TGFA: Transforming growth factor-*α*
TGFB: Transforming growth factor-*β*
Th17: T helper 17 cells
TLR4: Toll-like receptor 4
TNFA: Tumor necrosis factor-*α*
TPA: Tetradecanoyl-phorbol-13-acetate
TRKA: Receptor of nerve growth factor A
VEGF: Vascular endothelial growth factor
WTACE2: Pharmacological inhibitor of tumor necrosis factor-alpha-converting enzyme (TACE)
*α*SMA: *α*-Smooth muscle actin.
